# Pixel 5 Versus Pixel 9 Pro XL—Are Android Devices Evolving Towards Better GNSS Performance?

**DOI:** 10.3390/s25144452

**Published:** 2025-07-17

**Authors:** Julián Tomaštík, Jorge Hernández Olcina, Šimon Saloň, Daniel Tunák

**Affiliations:** 1 Department of Forest Resources Planning and Informatics, Faculty of Forestry, Technical University in Zvolen, T.G. Masaryka 24, 96001 Zvolen, Slovakia; 2 Department of Cartographic Engineering, Geodesy and Photogrammetry, Universitat Politècnica de València, Camino de Vera s/n, 46022 Valencia, Spain

**Keywords:** global navigation satellite systems, raw GNSS data, smartphones, signal quality, positioning accuracy

## Abstract

Smartphone GNSS technology has advanced significantly, but its performance varies considerably among Android devices due to differences in hardware and software. This study compares the GNSS capabilities of the Google Pixel 5 and Pixel 9 Pro XL (Google LLC, Mountain View, CA, USA) using five-hour static measurements under three environmental conditions: open area, canopy, and indoor. Complete raw GNSS data and the tools used for positioning are freely available. The analysis focuses on signal quality and positioning accuracy, derived using raw GNSS measurements. Results show that the Pixel 9 Pro XL provides better signal completeness, a higher carrier-to-noise density (C/N0), and improved L5 frequency reception. However, this enhanced signal quality does not always translate to superior positioning accuracy. In single-point positioning (SPP), the Pixel 5 outperformed the Pixel 9 Pro XL in open conditions when considering mean positional errors, while the Pixel 9 Pro XL performed better under canopy conditions. The precise point positioning results are modest compared to the current state of the art, only achieving accuracies of a few meters. The static method achieved sub-decimeter accuracy for both devices in optimal conditions, with Pixel 9 Pro XL demonstrating a higher fix rate. Findings highlight ongoing challenges in smartphone GNSS, particularly related to the limited quality of signals received by smartphone GNSS receivers. While newer devices show improved signal reception, precise positioning remains limited. Future research should explore software enhancements and the use of various external correction sources to optimize GNSS accuracy for mobile users. Generally, a shift from research to user-ready applications is needed.

## 1. Introduction

The integration of global navigation satellite system (GNSS) receivers into smartphones and other “smart” devices provided the opportunity to determine the relatively accurate position in relation to a very wide range of users. This ability, formerly available only to specialists, enabled the creation of an entirely new branch of software services, so-called Location-Based Services (LBSs) [1]. The main objective of this study is to evaluate whether the newer generation of Android smartphones offers measurable improvements in GNSS performance. To this end, we conduct a systematic comparison between two Google Pixel models, Pixel 5 (2020) and Pixel 9 Pro XL (2024), using static five-hour measurements under three environmental conditions: open sky, canopy, and indoor. The assessment focuses on both signal quality (carrier-to-noise density, observation completeness, cycle slips, multipath) and positioning accuracy across multiple methods: internal solution, single-point positioning (SPP), precise point positioning (PPP), and relative “static” positioning.

Since the introduction of the first GPS-enabled phone, the technology has undergone significant development. One of the most significant advancements has been the shift to multi-constellation, multi-frequency receivers. For Android devices, a very promising change was the availability of recording raw GNSS data, which was enabled in 2016. This functionality is currently mandatory for all devices running Android 10 or higher [2]. However, not all devices provide the Accumulated Delta Range parameter, which is crucial for the determination of carrier-phase observables. Thus, many devices are restricted only to code measurements, which limits the applicability of such raw data. But rather than a “black-box” internal GNSS solutions, where the device provides only the final coordinates, the raw data provides the possibility to apply other positioning methods, formerly available only to devices of a higher grade. Researchers have tested the “classic” methods, such as single-point positioning (SPP) [3], differential GNSS (DGNSS) [4,5], real-time kinematic (RTK) [6,7], as well as relatively new approaches, including precise point positioning (PPP) [8,9] and the utilization of Galileo High Accuracy Service [10]. A significant group of experiments utilized smartphone functionalities, which are not typical for standard GNSS receivers, such as cloud computing [11] and sensor fusion [12,13,14]. Besides the positioning performance, multiple studies have been aimed at qualitative characteristics of received signals. These include multipath effect, carrier-to-noise density (C/N0), satellite availability, and other [15,16,17].

The majority of studies focused on raw GNSS data from Android smartphones takes place under conditions ideal for signal reception. This is logical as during the initial test of new capabilities, the removable external sources of errors should be eliminated. However, the typical use of smartphones often includes places with adverse effects, mostly comprising blocking and multipath propagation of GNSS signals. Typical examples of such conditions are urban areas with high buildings (“urban canyons”) and areas with trees’ canopy (parks, forests). The negative effects are more pronounced on smartphone GNSS receivers than on higher-grade devices [18,19]. To mitigate them, multiple approaches have been proposed, including, e.g., integration with inertial measurement unit (IMU) [13] and 3D maps [20].

Any generalization of the GNSS performance for Android devices is very complicated due to the high diversity in hardware and software configurations. Even though the exact number is very hard to determine, there are hundreds of new types of Android devices every year. This leads to frequent outcome that the majority of studies uses only the devices from companies with significant market share [21], or devices with specific relation to GNSS measurements. A typical example is the Xiaomi Mi 8 phone, the worldwide first phone with dual-frequency GNSS receiver, which was given significant attention throughout the Android GNSS research community [22,23,24]. If multiple devices are compared, this is often a selection of models currently available to authors, without any intention to describe trends in the industry. One of the exceptions is the study from Szot et al. [25], where the authors compare several consecutive models of Samsung Galaxy phones. A notable result from the study is that the newer smartphone hardware doesn’t necessarily bring better GNSS performance. On the other hand, Odolinski et al. [26] compared several flagship smartphones, including three consecutive generations of Google Pixel phones, and reported similar positioning performance in the RTK positioning. In our study, we compare a Pixel phone of the current generation to the several generations older phone of the same family, to assess the evolution of GNSS performance.

Another constraint when evaluating the smartphone GNSS characteristics is the fact that the manufacturers very rarely declare specific information on GNSS-related hardware. Thus, even if some devices use the same GNSS chipsets, confirming this is difficult, which in turn further complicates any hardware-related generalization. However, the lack of official information is by a high margin substituted by a database maintained by Dr. Sean Barbeau, based on data from the GPSTest application [27], currently comprising of more than 7000 individual hardware/software (OS version) configurations [28]. Since 2016 Google has been providing a Pixel series of smartphones, which could be considered a reference design for Android devices. However, based on the aforementioned, it is only possible to recognize them as a reference from a software point of view only.

Since the introduction of the possibility to record raw GNSS data by Android smartphones, there were some specific problems complicating the precise positioning by smartphones. Generally, such data are of a lower quality than the data originating from devices of a higher grade, therefore it is needed to carefully adjust the processing software to deal with the data. This includes e.g., adjusting post-processing thresholds [6,29], which would normally cause discarding majority of the data, thus rendering them useless. Another, currently already solvable issue, is so-called duty-cycling. This OS feature, aimed at saving battery life, causes periodic interruptions of the GNSS receiver activity. These interruptions are transferred into the data, where the continuousness is crucial to the positioning, especially when carrier-phase observations are employed. Firstly, it was possible to turn off the duty-cycling via developer options under OS settings. However, since Android 12 this option can be included directly in the data-recording software [30]. Another group of issues is related to the smartphone GNSS antenna. Due to cost and space constraints, the antenna is very simple compared to antennas of higher-grade devices. Such simplicity causes significant proneness to signal interruptions, lower signal strength (up to 10 dBHz compared to survey-grade antennas [31]) and multipath [17,32]. The precise positioning by smartphones is further complicated by the unknown location of the antenna phase center. This was addressed by several research teams, where the phase center was determined using biases/shifts based on longer observation periods [16] or even robotic calibration [33]. However, the results are applicable only on the particular device, or with some degree of generalization to a specific model. Since Android 11, the antenna characteristics (phase center offset and variations) could be accessed via the GnssAntennaInfo class [34]. But according to the abovementioned database [28] this information is provided only for a very limited portion of smartphone models.

This aligns with the main aim of our study: to explore generational improvements in GNSS signal reception and positioning outcomes in smartphones using raw data and consistent testing conditions.

## 2. Materials and Methods

### 2.1. Data Acquisition

To acquire the data, we used two smartphones—Google Pixel 5 and Google Pixel 9 Pro XL. According to documentation, the GNSS hardware year of the devices is 2018, resp. 2023. None of the tested devices has a dedicated GNSS chip, unlike e.g., Google Pixel 6, 7, 8 with a Broadcomm BCM4776 chip (Broadcomm, Palo Alto, CA, USA). For Pixel 5, the GNSS positioning capabilities are part of the Qualcomm Snapdragon 765G system-on-chip (SoC) (Qualcomm, San Diego, CA, USA), while for the Pixel 9 Pro XL the crucial component is a Samsung Exynos 5400 modem (Samsung, Gyeonggi-do, Republic of Korea). Both the devices use multi-constellation (all global systems), dual-frequency receivers. A significant difference is in the reception of BeiDou signals—while the Pixel 5 phone uses only B1I signal, the Pixel 9 Pro XL is also capable of receiving the B1C and B2a signals (see Table 1).

**Figure 1:**
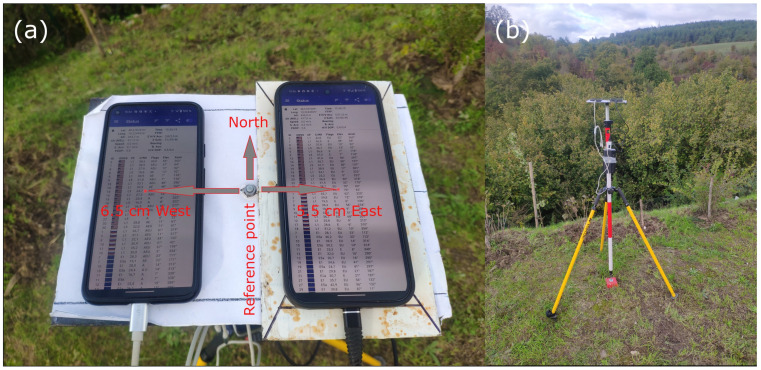
Placement of the tested devices (**a**) and overall hardware setup (**b**).

The devices were placed on a simple steel ground plane. The beneficial effect of such a plane on the quality of smartphone GNSS measurements was proved in a previous study [35]. Surface of the plane is white (using paint or glued paper) to avoid overheating during sunny days and to enable markings used for stable placement of the devices. The plane was placed on a tripod at a 1.4 m height (Figure 1b). Before the measurement, the ground plane was oriented to match the longer axis of the devices with the North–South direction. The center of the Pixel 5 phone was shifted 6.5 cm to the West from the reference point, while for the Pixel 9 Pro XL the shift was 5.5 cm to the East (Figure 1a). These shifts were considered during the positioning tests, and the errors in the E-W direction were correspondingly adjusted. We did not test nor consider the phase centers of the antennas; thus all positions are related to the center of the particular device. To maintain a longer observation period, the devices were powered using a powerbank.

**Figure 2:**
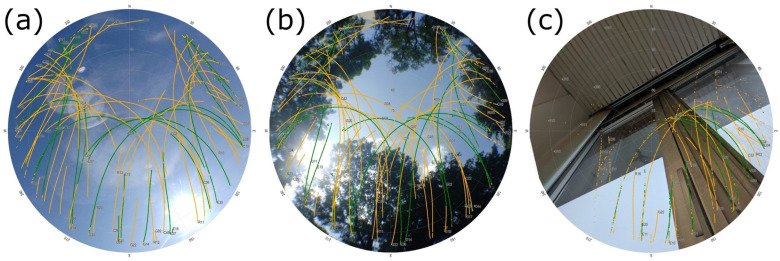
Skyplots illustrating the conditions involved in the experiment: “open” (**a**), “canopy” (**b**), and “indoor” (**c**). Satellite tracks are taken from Pixel 9 Pro XL measurements.

To evaluate the influence of differing conditions on the GNSS performance, all measurements were repeated on three points with differing conditions (Figure 2). The “open” point represents nearly ideal conditions with no obstructions over a 10° elevation angle. This point, where we did not expect increased effects adverse for signal reception, can be considered a comparison basis for other points with more complicated conditions. The “canopy” point is located in a park, surrounded by a mixture of coniferous and deciduous trees. The canopy is interrupted by a north-facing gap, approximately between 45° and 90° elevation angle. The measurement was conducted in autumn, thus we estimate 20% foliage loss in the time of the measurement. On this point, we expected a significant influence of the multipath effect and short-term interruptions of the signal reception due to the vegetation. The “indoor” point was placed near a window on the second floor of a four-story university building. The direction of the relatively undisturbed signal reception is South-East. Here we expected the main influence of total signal blockage caused by the building.

The reference coordinates of the “open” point were acquired using static GNSS measurements, for the “canopy” and “indoor” point the GNSS positioning was combined with the Topcon GPT9000 total station (Topcon, Tokyo, Japan) measurements. Due to national standards, the coordinates are in the ETRS89 (ETRF2000, epoch 2008.5) frame. For differential positioning (static method) we used this reference frame, while for the single-point positioning (SPP), precise point positioning (PPP) methods and device’s internal solution we transformed the coordinates into the ITRF2020 frame using the EUREF transformation service [36]. The reference coordinates of test points in both the ITRF2020 and ETRF2000 are included in a publicly available dataset [37].

Two applications were used to record the GNSS data—GEO++ RINEX logger [38] and GnssLogger App [39]. The GEO++ RINEX logger 2.1.8 was used to record the data in the RINEX format, while the GnssLogger 3.1.0.3 was used to record device’s internal solution in the NMEA format. Both the applications run simultaneously on both tested devices. The data was recorded for five hours in approximately the same time between 8:00 and 14:00 UTC. The specific dates of the measurements were 10 October 2024 for “indoor”, 15 October 2024 for “open”, and 29 October 2024 for “canopy” conditions. The data were not manipulated in any way (e.g., filtered) prior to the analyses.

Experimental data including RINEXes from base and rover, reference coordinates and processing configuration are available via Mendeley Data [37].

### 2.2. Quality Control (QC)

The GNUT/Anubis 3.5 software [40] was used to evaluate basic qualitative characteristics of received signals. The software analyses multi-constellation, multi-frequency raw data saved in RINEX files. We used RINEX files acquired using GEO++ RINEX logger. The software runs on a command line. The QC mode was set to “full”, with all the other parameters left default according to the software manual [41]. The only exception was the qc:sat_rec=false parameter, thus the calculation of the expected observations took only the satellites able to provide the particular signal into account—e.g., only GPS satellites capable of providing the L5 signal, instead of all trackable GPS satellites.

The mean values of the have/expected observations ratio, carrier-to-noise density (C/N0), cycle slips, and multipath (MP) were extracted from the structured text files in the GNUT/Anubis native format (.xtr) according to the individual signals. In the case of have/expected observations ratio, the results included code as well as carrier-phase observations. The absolute number of cycle slips was divided with the number of present observations, thus resulting in relative expression of this measure. In the cycle slips analysis, also three other sources of discontinuities were included in the total count—due to missing epochs, due to missing satellites and due to missing signal when considering two consecutive epochs. A closer explanation can be found in the GNUT/Anubis manual [41]. The mean values (calculated from all available epochs) were used to compare the devices and conditions involved.

### 2.3. Positioning

In the positioning experiment, we used four positioning methods—“internal”, “single-point positioning” (SPP), “precise point positioning” (PPP) and “static”.

“Internal” solution is the solution provided directly by the phone hardware and software, without any raw data postprocessing. It is impossible to directly influence this solution, but this is the position which the average smartphone GNSS user gets standardly. These solutions were recorded in the NMEA format, using the GNSSLogger application. The positions were displayed using the RTKPLOT module of the RTKLib library. The devices and conditions were subsequently compared using the mean horizontal errors and relevant variability parameters.

The postprocessing of the raw GNSS observations was conducted in the Demo5 b34k fork of the RTKLib software, Ver. 2.4.2 [42]. This version is adapted to processing the data from low-cost GNSS receivers. The “Single” method in the RTKLib refers to single-point positioning (SPP), where the position is determined using solely the pseudoranges acquired using one receiver. Regarding the configuration, we used default settings, with 10° elevation mask and inclusion of all global systems (GPS, GLONASS, Galileo, BeiDou). Neither regional systems (QZSS, IRNSS) nor satellite-based augmentation systems (SBAS) were used. The precise point positioning method was conducted using the CSRS-PPP service. Due to sufficient time between the measurements and the processing, NRCan/IGS final ephemeris were used. The “static” method is a differential method involving both code and carrier-phase observations. In principle, it uses the Post-Processed-Kinematic (PPK) method approaches in the RTKLib software, where the position is estimated under the assumption that the receiver remains stationary throughout the observation period. Internally, this mode employs an extended Kalman filter with fixed position dynamics, meaning that the position state is considered constant over time, allowing more robust resolution of carrier-phase ambiguities. Specifically, we used the “combined—no phase reset” filter type (filter type = 3 in RTKLib configuration) that fuses both the forward and backward solution (i.e., with increasing time and decreasing time) without resetting the phase in transition from forward to backward solution, which is standard in the default “Combined” filter.

**Figure 3:**
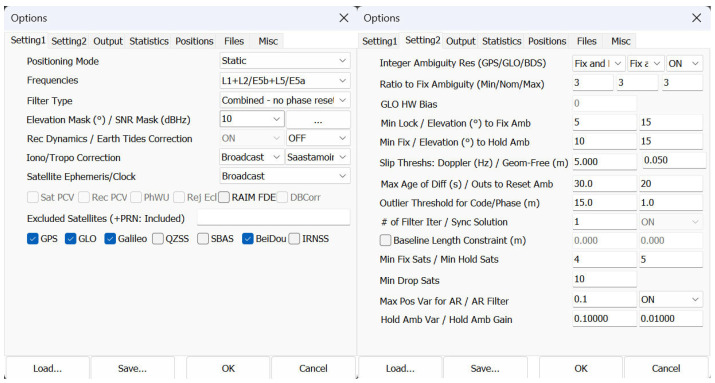
Basic setting used for the static method in the RTKLib software.

Although full mathematical derivation of the Kalman filter is outside the scope of this study, its principle is based on iterative state updates using measurement innovations and associated covariances. These mathematical foundations are fully documented in RTKLib Demo5 manual [43]. For reproducibility, we provide the complete configuration file used for RTKLib (“phone.conf”) as part of the shared dataset. This file contains all parameters used, including the elevation mask, satellite weighting, dynamic model settings, and ambiguity resolution thresholds, which were fine-tuned for smartphone raw GNSS data as recommended in [28]. Basic settings are summarized in Figure 3. These were supplemented with detailed settings aimed at dealing with the poor-quality smartphone data, including: the C/N0 mask set to 24 for both frequencies, code/phase error ratio 1500 for L1 (default 300), carrier-phase error (both base and elevation dependent term) set to 0.006 m (default 0.003 m), and carrier-phase bias 0.001 m (default 0.0001 m).

The corrections were provided by the national SKPOS service, where we used the data from the nearest continuously operating reference station (CORS) for each point. The baseline was 9.6 km long for the “open” point, while 2.7, respectively 2.8 km for the “canopy” and “indoor” point [29].

The results were compared to the reference coordinates. For the absolute positioning methods (internal, SPP and PPP) we used the ITRF2020 frame. For the “static” method, the ETRS89 (ETRF2000 epoch 2008.5) was used, as the national CORS network provides data in this frame.

The mean horizontal error (*HE*) served as the primary basis for comparing devices, methods, and conditions. It was calculated using Equation (1) by averaging the individual horizontal errors from all available solutions (derived from Easting and Northing errors), which enabled a detailed statistical analysis.

(1) $$HE=\frac{\sum \sqrt{dE_{i}^{2}+dN_{i}^{2}}}{n}$$

*dE_i_* and *dN_i_* represent Easting and Northing errors for each epoch, n is the number of available epochs.

Additionally, for each measurement, we report the horizontal error of the final averaged position (*AP*) calculated from mean coordinate errors as follows

(2) $$AP=\sqrt{{\bar{dE}}^{2}+{\bar{dN}}^{2}}$$

where $\bar{dE}$ and $\bar{dN}$ are average Easting and Northing coordinate errors.

For height assessments, the mean vertical error (*VE*) was used as the corresponding comparative measure.

## 3. Results

According to the Methodology, the results are divided into two main Section 3.1. Quality Control and Section 3.2.

### 3.1. Quality Control

In this section, we summarize the mean values of signal quality measures (have/expected observations ratio, cycle slips, C/N0, multipath) calculated according to device, conditions and signals, using all the available epochs (~18,000).

#### 3.1.1. Have Versus Expected Observations

**Figure 4:**
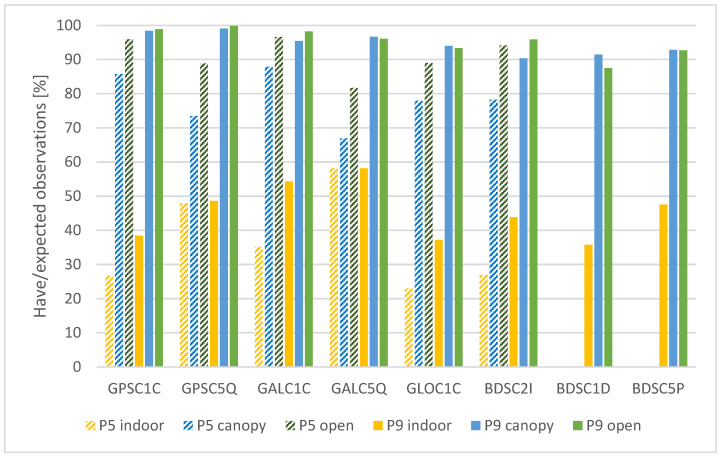
Have/expected ratio for code observations according to devices, conditions and signals.

**Figure 5:**
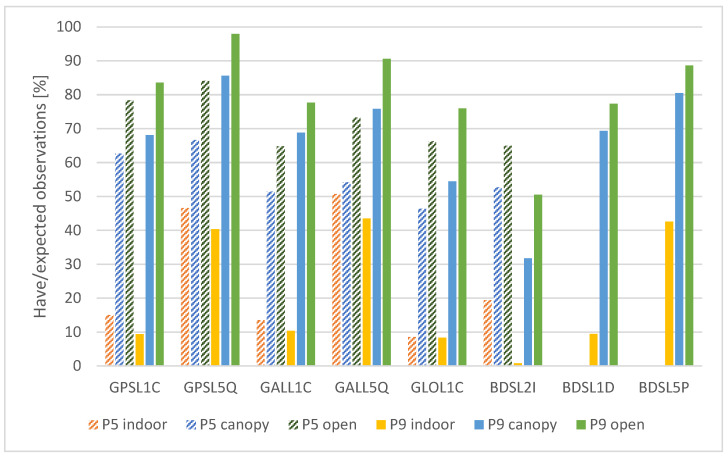
Have/expected ratio for carrier-phase observations according to devices, conditions and signals.

As a first measure, we evaluated a ratio between have and expected observations, taking only the elevation angles over 10° into account. The “have” observations represent the observations, which are actually recorded in the raw GNSS data. The “expected” observations are determined using approximate location of the measurement, time and predicted satellite trajectories. The summary of the results is divided into two separate parts, including code observations (Figure 4) and carrier-phase observations (Figure 5).

The differences between the measurement conditions are clearly visible. For the Pixel 5 phone, these copy the expected order (“indoor”-“canopy”-“open”) in all cases. The ratio of present observations averages at about 23% under “indoor” conditions and at 85% for the “open” area. Surprisingly, for the Pixel 9 Pro XL phone there are multiple cases where the ratio between the present and expected observation is the highest under the “canopy” conditions, although besides the BDSC1D the differences are minimal. However, the “canopy” and “open” values are much closer to each other than for the Pixel 5, averaging at ~95% with exception of BeiDou signals, which still exceed 90% in most cases. The implementation of dual-frequency BeiDou measurements can be considered one of the main technological achievements when comparing the current Pixel generation to the older phone. The “completeness” of the newly implemented BeiDou observables is only marginally lower than for the other systems.

General between-device comparison for code observations shows that the newer device provided a higher have/expected ratio with differences ranging from ones to tens of percent. The only exceptions were the GPS and GAL L5 signals for the “indoor” conditions, where the values are approximately the same for both the devices. The highest differences between the devices can be seen under suboptimal (“canopy” and “indoor”) conditions.

Regarding the carrier-phase observations, the have/expected ratio is significantly lower than for the code observations. In all tested cases the order of the conditions is from the worst (“indoor”) to the best (“open”). The ratio is higher for the Pixel 9 Pro XL phone in most cases apart from indoor conditions and BDS L2I signal. The device apparently prefers the BeiDou L1D and L5P signals over the L2I. The average ratio under the optimal conditions is 72% for the Pixel 5, while 80% for the Pixel 9 Pro XL. The other visible trend is the preference of L5 signals over L1 signals. Under all tested conditions, the have/expected ratio was higher for the L5 signals for all systems providing dual-frequency observations (GPS and GAL for Pixel 5, GPS, GAL and BDS for Pixel 9 Pro XL).

#### 3.1.2. Cycle Slips

**Figure 6:**
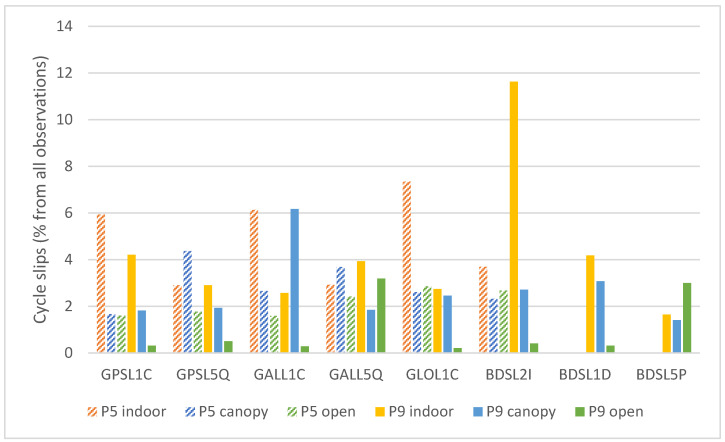
Ratio between cycle slips and present carrier-phase observations according to devices, conditions and signals.

The cycle slips represent discontinuities in the signal tracking and jumps of integer number of wavelengths [44]. The cycle slip occurrence complicates the ambiguity resolution and deteriorates the results of carrier-phase based methods. The rate of the occurrence of cycle slips is summarized in Figure 6.

Generally, the occurrence of discontinuities is the highest under nonoptimal conditions, but there are some exceptions. For the Pixel 9 Pro XL and Galileo L5Q signal, the rate of cycle slips is higher under “open” conditions compared to the “canopy” conditions. For BeiDou L5P signal it is even higher than for “indoor” conditions. Excluding these two cases, the cycle slip rate for the Pixel 9 Pro XL in optimal conditions is rather low, averaging at ~0.35%. For the Pixel 5, the average cycle slip rate under optimal conditions is around 2.15%. For this device, there are two cases, where there is a higher number of cycle slips for “open” conditions than for “canopy” conditions (GLONASS L1C and BeiDou L2I). In two cases (GPS L5Q and Galileo L5Q) the rate is higher for “canopy” than for “indoor” conditions.

The highest cycle slips rate (11.63%) is reported for the Pixel 9 Pro XL under “indoor” conditions, considering the BeiDou L2I signal. However, this case illustrates the necessity to interpret the cycle slips measures in accordance with an absolute number of present observations. There were only 43 BeiDou L2I observations (out of ~18,000 epochs) in this particular case, with 5 cycle slips identified.

The closer analysis of the discontinuities in the observations showed that rather than the real cycle slips (jumps in the carrier-phase observations), the major reason is the interruptions of signal reception when considering two adjacent epochs.

**Figure 7:**
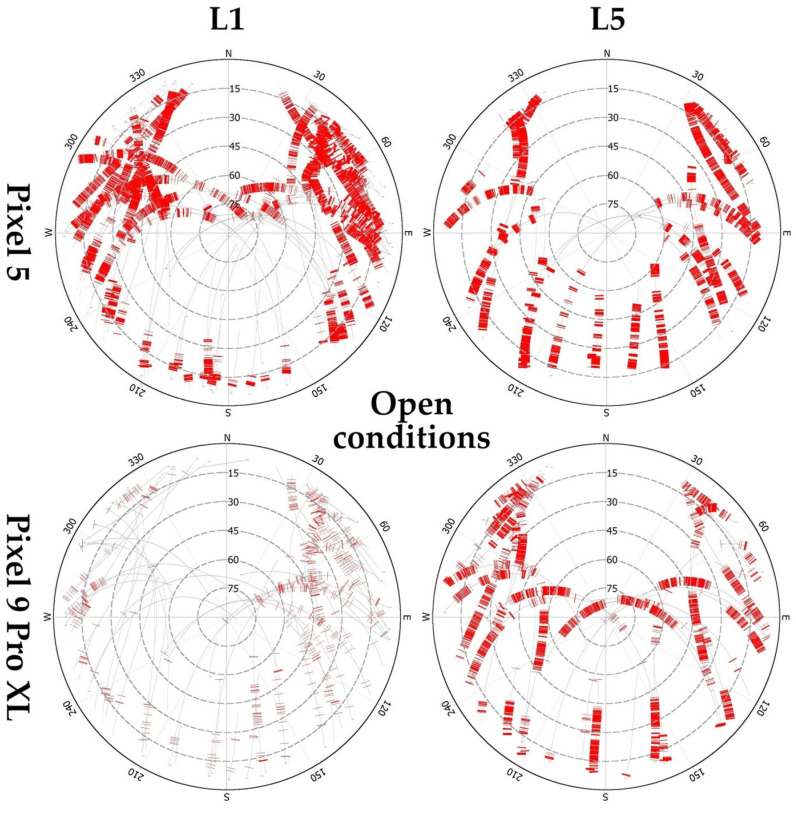
Skyplots with cycle slips occurrence according to devices and signal frequencies under “open” conditions. Red ticks represent “Lost Lock Indicator” (LLI) flags extracted from RINEX files. Skyplots for canopy and indoor conditions are provided as Appendix A.

The spatial distribution of cycle slips, specifically Lost Lock Indicator (LLI) flags from RINEX files is shown in Figure 7 (open conditions) and Appendix A (canopy and indoor). For Pixel 5, the L1 cycle slips are concentrated in the northern hemisphere and elevations below 30°, while for L5 only the elevations over 60° are relatively cycle-slip free. For Pixel 9 Pro XL, LLI flags for L1 signals are visibly less dense and concentrated in the eastern hemisphere. For L5 there is only a relatively small window with continuous reception of signals between azimuths ~130° and 210° and elevations between 40° and 75°. The cycle slips for Pixel 5 under canopy conditions are relatively dense even in open parts, in contrary to the Pixel 9 Pro XL, which provided a more continuous reception in these gaps. Interestingly, under indoor conditions the lower presence of cycle slips for both devices and frequencies can be seen in the southwest unobstructed portion of the skyplot.

#### 3.1.3. Carrie-to-Noise Density

**Figure 8:**
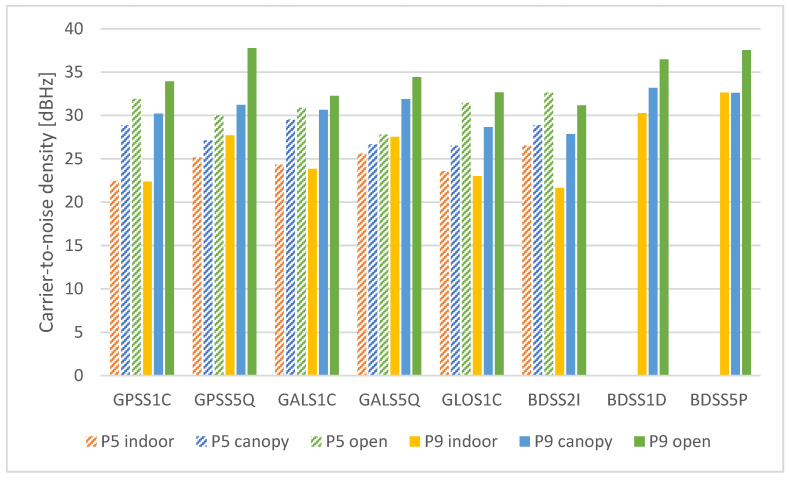
Mean carrier-to-noise density according to devices, conditions and signals.

The signal-to-noise density (C/N0) is the most commonly used measure of signal strength. The average values according to devices and conditions are shown in Figure 8.

The tested conditions had an unambiguous influence here. In all cases, the C/N0 is the highest under the “open” conditions, while the lowest for the “indoor” conditions. For Pixel 9 Pro XL, the transition from “open” to “canopy” caused a C/N0 decrease from 1.6 to 6.5 dBHz depending on particular signals, while between “open” and “indoor” conditions the decrease is from 4.9 to 11.6 dBHz. For Pixel 5, these values range are between 1.1 and 4.9 dBHz (“open” versus “canopy”) and from 2.2 to 9.5 dBHz (“open” versus “indoor”).

The Pixel 9 Pro XL provided a higher C/N0 under “canopy” and “open” conditions, with one exception being the BeiDou L2I signal. The highest differences can be seen for L5 signals, where under the “open” conditions the Pixel 5 provided a C/N0 lower by 7.72 (GPS L5) and 6.63 dBHz (Galileo L5).

Regarding the BeiDou III signals (L1D and L5P), which are specific for the Pixel 9 Pro XL phone, their carrier-to-noise density was among the highest in all tested cases. Even their values under “indoor” conditions are very close to the results achieved by Pixel 5 phone in optimal conditions.

**Figure 9:**
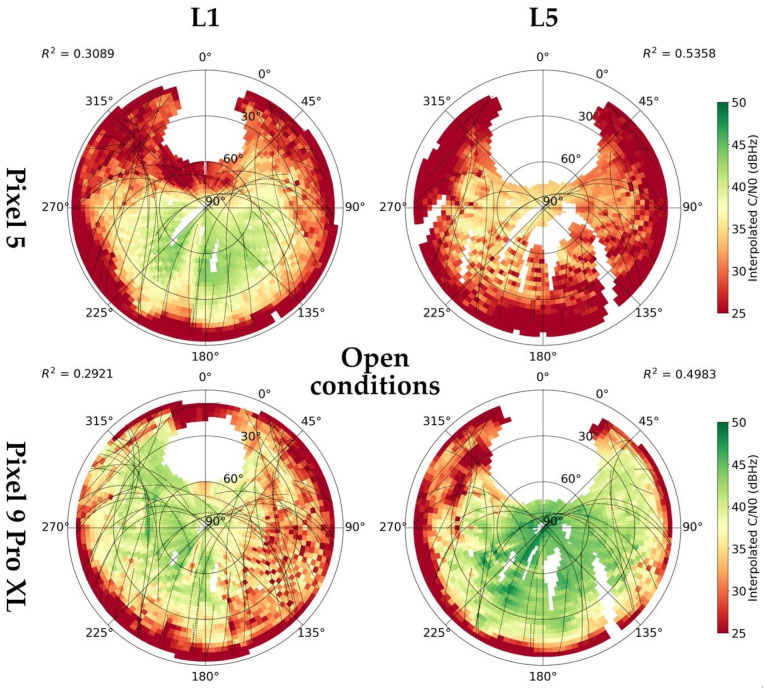
Skyplots with interpolated values of carrier-to-noise density (C/N0) according to devices and signal frequencies under “open” conditions. Coefficients of determination between C/N0 and elevation angle. Skyplots for canopy and indoor conditions are provided as Appendix B.

Figure 9 shows interpolated C/N0 values under open-sky conditions. The values were interpolated for 3 × 3° cells, 9° from the satellite trajectories at most. The pattern for Pixel 5 agrees with the distribution of cycle slips; the highest L1 C/N0 values are on the southern hemisphere, while L5 C/N0 is generally lower with a maximum over elevation of 60°. An irregular loss in L1 signal strength is evident in the East direction for the Pixel 9 Pro XL. In contrast with Pixel 5, the L5 values are high even considering lower elevations. Under canopy conditions (Appendix B), higher C/N0 values are shifted towards higher elevations and parts without obstructions. However, the L5 reception for Pixel 9 Pro XL is relatively good even through the canopy cover. Interestingly, under indoor conditions, the higher C/N0 values are concentrated in parts with high occurrence of cycle slips. There is also an evident occurrence of no-line-of-sight signals.

#### 3.1.4. Code Multipath

**Figure 10:**
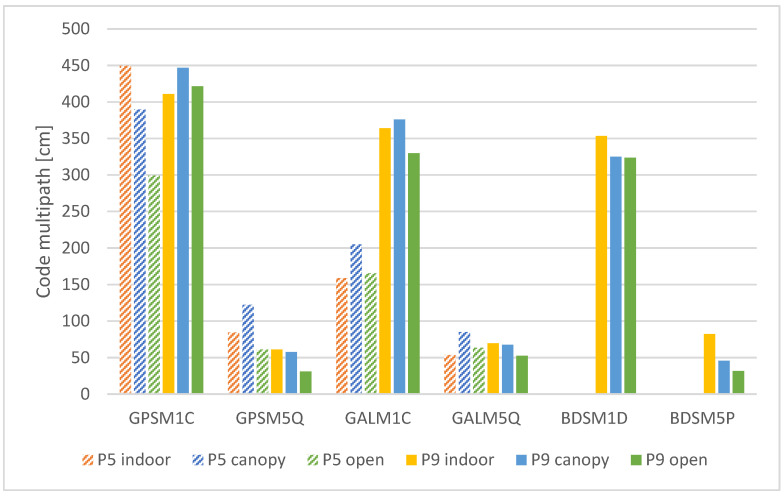
Mean code multipath according to devices, conditions and signals.

The code multipath (MP) is calculated as a linear combination of code and carrier-phase observations. Therefore, it needs uninterrupted, mutual reception of dual-frequency code and phase observables. This was proven to be an issue for smartphone receivers, especially under nonoptimal conditions., e.g., under the “indoor” conditions, the presented MP is calculated only based on 2–4 satellites observations, which cannot be considered robust and could be prone to extremes. Despite this limitation, the code multipath values are summarized in Figure 10.

The only clear statement, based on the presented results, is that the multipath for L5 signals is significantly lower than that for L1 signals. The L1 multipath is in the order of meters, while the L5 multipath is in the order of decimeters. Regarding the external conditions, there are multiple cases, when the multipath for “open” and “canopy” is higher than for “indoor” conditions. We explain this by the already mentioned insufficient number of mutual, dual-frequency code/phase observations for “indoor” conditions.

Comparison of the devices is possible only for the GPS and Galileo L1 and L5 signals as the Pixel 5 does not provide dual-frequency measurements for BeiDou. The Galileo L1 signals received by the Pixel 5 phone were significantly less influenced by the multipath compared to the Pixel 9 Pro XL under all tested conditions. For other comparable signals, the distribution of mean errors is rather random and does not show any general trend.

### 3.2. Positioning

This section provides an overview of the positioning results achieved using internal, SPP, PPP and static method according to devices and conditions. All the available solutions were used to determine the statistical characteristics.

#### 3.2.1. “Internal” Solution

**Figure 11:**
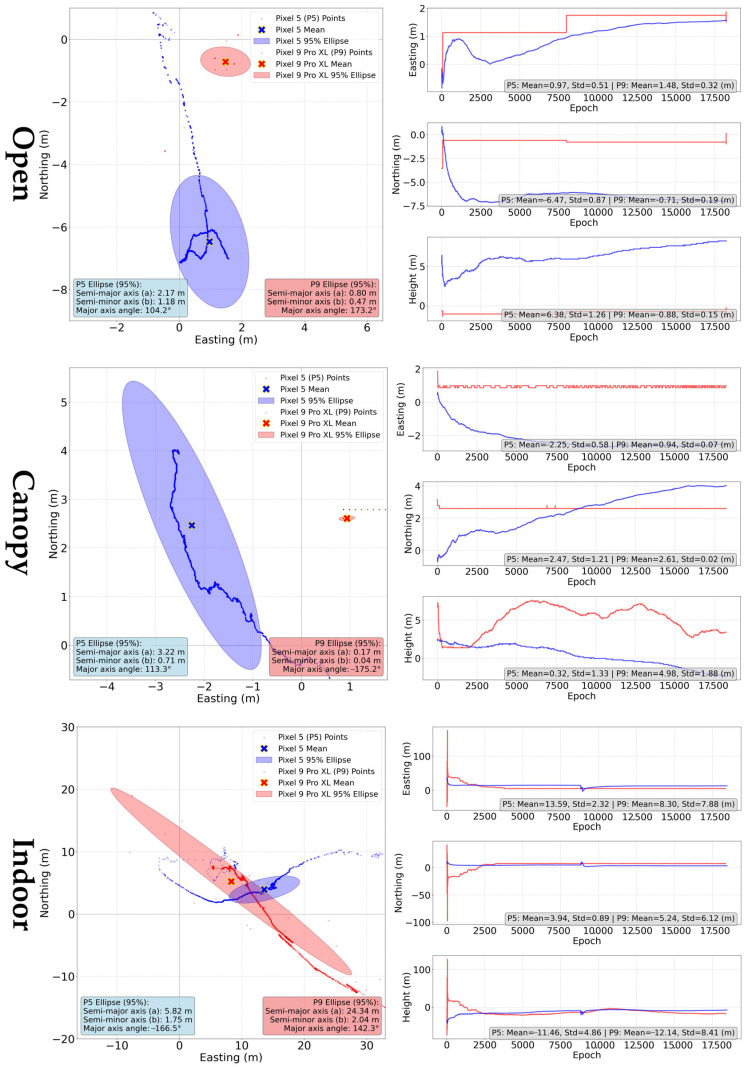
Ground tracks and coordinate errors of internal positioning solutions for Pixel 5 (blue) and Pixel 9 Pro XL (red) according to measurement conditions. Ground track plots were scaled to fit 95% ellipses, higher coordinate outliers can be identified in E, N, U subplots.

The positions were extracted from NMEA file and compared according to devices and conditions. The ground tracks and errors in the cardinal directions (Figure 11) show a significantly different behavior of the devices in relation to the long-term evolution of the internal solution while the device is static.

For Pixel 5, the solution moves continuously over time. A negative trend observed in the results is that after a short-term initial improvement, the solution accuracy deteriorates. This is especially visible under “open” and “canopy” condition. In the “indoor” conditions, this trend disappears in the high values of errors, reaching several tens of meters rather than ones of meters for the other conditions. The Pixel 9 Pro XL behaves significantly differently, when after an initialization period the coordinates change only in small incremental steps and tend to maintain the same value for a long time. Thus, a particular point in the graphic display of the solution can represent thousands of the solutions with the same coordinates. With deteriorating conditions, this trend disappears firstly for the height coordinate and in the worst condition also for horizontal coordinates.

As depicted in Table 2, the analysis of mean horizontal errors indicates that the Pixel 9 Pro XL generally exhibits lower errors compared to the Pixel 5, particularly in open environments where its accuracy is significantly improved. The vertical error patterns also differ, with the Pixel 9 Pro XL demonstrating less variability under open and canopy conditions, but slightly higher deviations indoors. This suggests that the performance of each device is influenced by environmental factors and specific device behavior.

The errors of the average position are very close to the mean horizontal errors, with the exception of Pixel 9 Pro XL results under indoor conditions.

#### 3.2.2. “Single” Method

Single-point positioning (SPP) is an absolute positioning method, which uses only the code measurements to determine the position.

**Figure 12:**
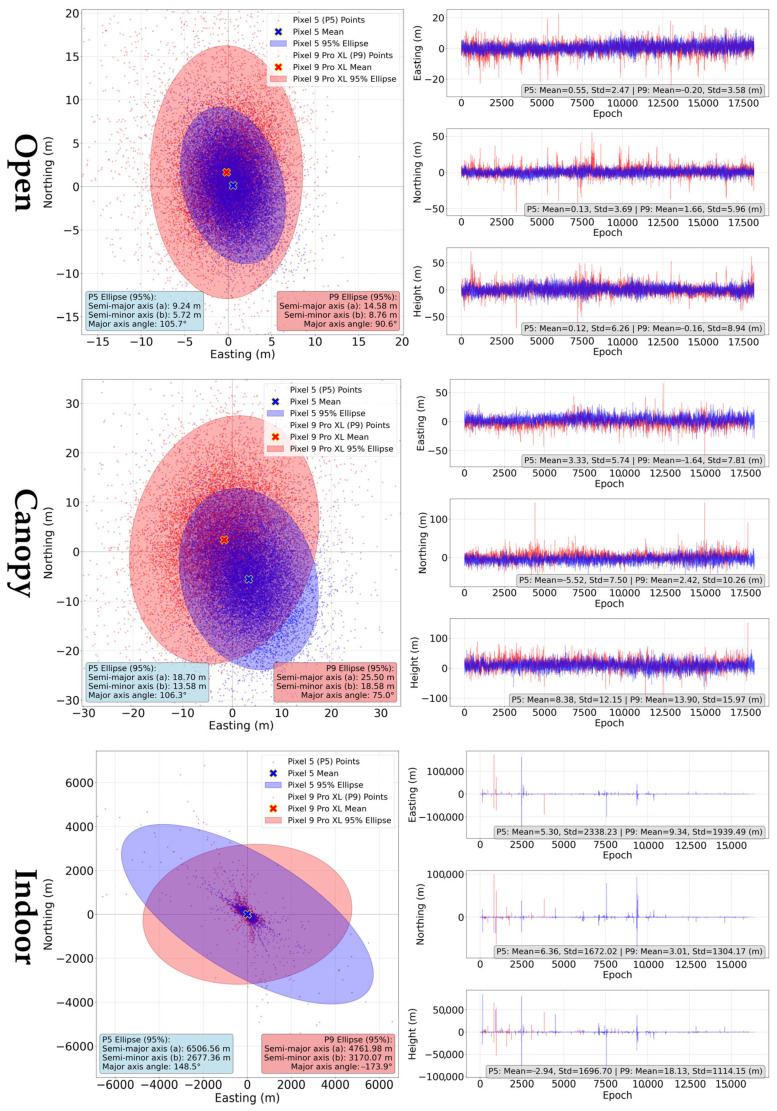
Ground tracks and coordinate errors of SPP method for Pixel 5 (blue) and Pixel 9 Pro XL (red) according to measurement conditions. Ground track plots were scaled to fit 95% ellipses, higher coordinate outliers can be identified in E, N, U subplots.

Already visual interpretation of the results summarized in Figure 12, supported by comparison of standard deviations, shows that the variability of the Pixel 9 Pro XL solutions was higher under both the “open” and “canopy” conditions, compared to the Pixel 5. The mean horizontal error of the Pixel 5 was lower under the “open” conditions, and also under the “canopy” conditions compared to the Pixel 9 Pro XL (Table 3). “Indoor” conditions are specific by a very high random component of the positioning errors. The standard deviations reach values over 2300 and 2800 m, while extremes exceeded tens of kilometers for both devices. Mean horizontal errors are highly influenced by these high values and are close to 200 m.

The “single” method is characterized by significant difference between mean horizontal errors (calculated as an average of horizontal errors for all epochs) and the error of the average position., e.g., in the aforementioned case of indoor conditions, the shift in the average position from reference position is 8.277 m (Pixel 5) and 9.814 m (Pixel 9 Pro XL), rather than ~200 m mean horizontal error. This visible difference is also present under open and canopy conditions.

#### 3.2.3. Precise Point Positioning

The PPP is an absolute positioning method, which uses code and carrier-phase data, and precise satellite ephemeris to augment the positioning results. In this experiment, we used CSRS-PPP service. The use of multiconstellation, dual-frequency data was limited by the fact that the service uses exclusively GPS and GLONASS data, and L1 and L2 frequencies. Thus, it was not possible to process the data using the ambiguity resolution (PPP-AR) method. Due to low quality, 30.78% epochs were rejected for Pixel 9 Pro “indoor” measurements, and 0.12% for Pixel 5 “canopy” measurement. The processing was unsuccessful for the Pixel 5 “indoor” data. In all other cases, 100% of available epochs were used. The results of the PPP method are summarized in Table 4.

**Figure 13:**
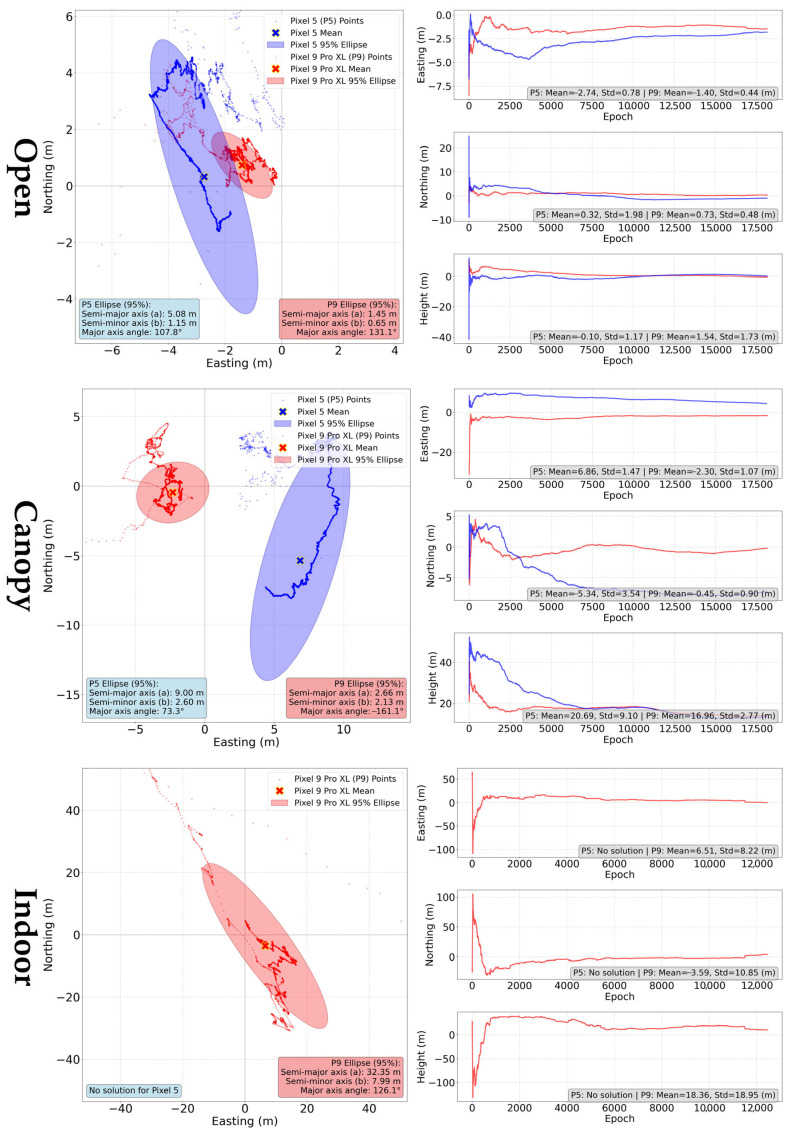
Ground tracks and coordinate errors of PPP method for Pixel 5 (blue) and Pixel 9 Pro XL (red) according to measurement conditions. Ground track plots were scaled to fit 95% ellipses, higher coordinate outliers can be identified in E, N, U subplots.

**Figure 14:**
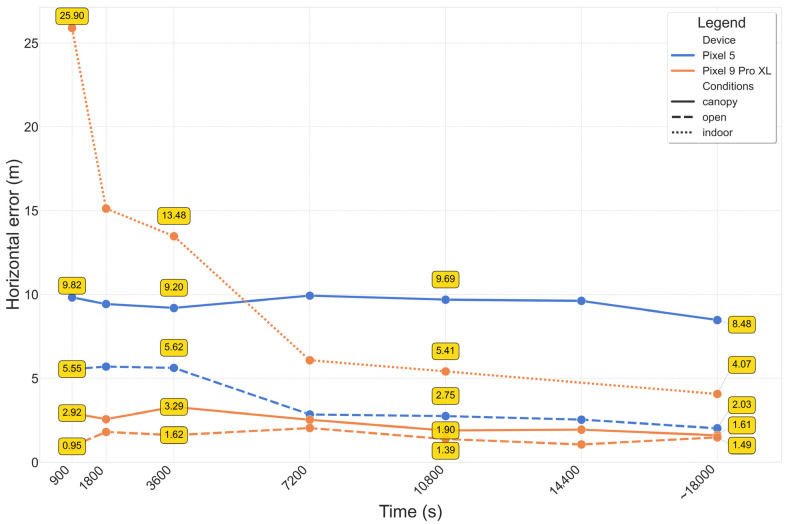
Evolution of PPP horizontal errors in time, according to device and conditions (no solution for Pixel 5 indoor). Annotated values after 15 min, one hour, three hours and at the end of the measurement (~5 h).

In this case, the Pixel 9 Pro XL outperformed the Pixel 5 in terms of horizontal error. Also, the variabilities for the Pixel 9 Pro XL are evidently lower in this case (Figure 13). A key characteristic of the PPP method is the convergence period, after which the solution should stabilize. Looking at Figure 11, it is complicated to determine an exact convergence period. The behavior varies significantly across different coordinates; for example under open conditions, the North and Up components vary relatively slightly already after a short time, but the East component “stabilizes” only after almost two hours. When using median horizontal error to mitigate the influence of high errors at the beginning of the processing, the median errors are 2.73 m (Pixel 5) and 1.46 m (Pixel 9 Pro XL) under open condition, while under indoor conditions the error decreases from 11.14 m (average) to 6.77 m (median) for the Pixel 9 Pro XL. Standardly, the PPP results are evaluated after the convergence period, which is highly device- and conditions-dependent in our case. The horizontal errors at the end of the measurement (Figure 14) are in all cases lower than the mean values due to the already mentioned influence of coarse errors during the initial phase of the processing.

#### 3.2.4. “Static” Method

The “static” method is a differential positioning method, which employs both code and carrier-phase measurements. In the case of Android smartphones, the device must be able to provide so-called Accumulated Delta Range (ADR) to determine the carrier-phase observables.

**Figure 15:**
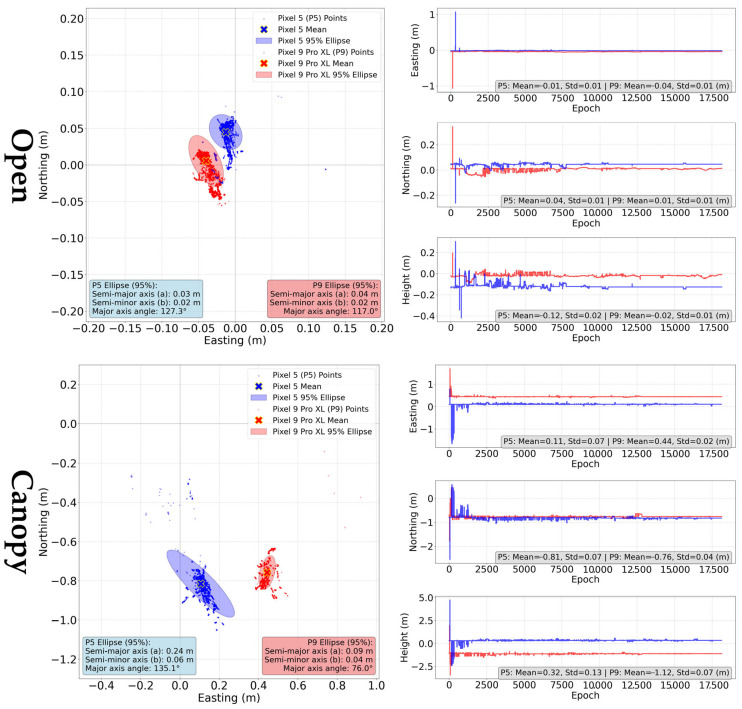
Ground tracks and coordinate errors of the “static” method for Pixel 5 (blue) and Pixel 9 Pro XL (red) according to measurement conditions. Green point represents the reference position.

Two specifics occurred while using the static method with our experimental data. Firstly, the calculation failed for both devices under the “indoor” conditions, resulting in “no solution” statement. Secondly, there is a visible shift in the solutions to the South for the “canopy” conditions (Figure 15). The possibility of a faulty reference was eliminated using repeated measurements.

Under optimal conditions (see Table 5), both the Pixel 5 and Pixel 9 Pro XL achieved sub-decimeter mean horizontal errors, with the Pixel 9 Pro XL performing slightly better at 0.042 m compared to 0.047 m for the Pixel 5. These errors are related to the centers of the devices and fall within the range of devices’ dimensions. For a more robust evaluation, the characteristics of the antenna phase center, which are unfortunately unknown, would be crucial. Therefore, the information on the variability would prove to be of higher importance than the mean values alone. The standard deviation for the Pixel 5 is 0.011 m, while it is 0.009 m for the Pixel 9 Pro XL. The inter quartile ranges (IQR) are 0.001 m and 0.005 m, respectively. Height errors were more pronounced for the Pixel 5, with a mean value of −0.120 m, whereas the Pixel 9 Pro XL exhibited a smaller deviation of −0.019 m. The Fix/Float ratio indicates a significant improvement in solution stability for the Pixel 9 Pro XL, with 82.1% of solutions being classified as Fix, compared to only 37.5% for the Pixel 5.

Under “canopy conditions” (see Table 6), a uniform bias in the N-S direction was observed, with values of 0.81 m for the Pixel 5 and 0.76 m for the Pixel 9 Pro XL. The horizontal errors were larger in this scenario, with the Pixel 9 Pro XL showing a mean horizontal error of 0.879 m, while the Pixel 5 registered 0.826 m. The standard deviations were 0.054 m (Pixel 5) and 0.025 m (Pixel 9 Pro XL), while the IQR was 0.015 m and 0.004 m. Height errors were also affected, with the Pixel 5 recording 0.323 m and the Pixel 9 Pro XL showing a greater deviation of −1.119 m. The Fix/Float/DGNSS ratio revealed that under canopy conditions, both devices relied more on float solutions, with the Pixel 9 Pro XL presenting a lower Fix ratio (13.4%) compared to the Pixel 5 (24.8%). In the case of static method, the differences between mean horizontal errors (HE) and errors of the average position (AP) were marginal, only in range of few millimeters.

#### 3.2.5. Statistical Summary

**Figure 16:**
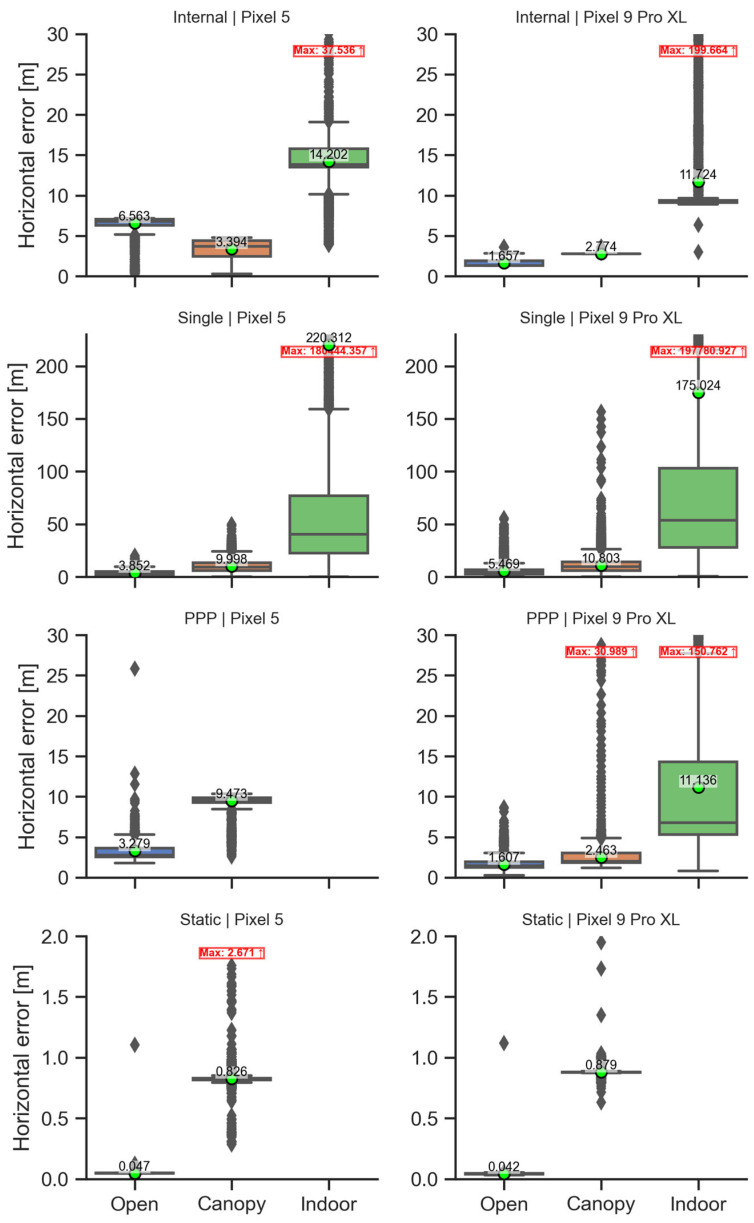
A summary of statistical characteristics of horizontal errors according to device, method, and condition. Mean (green circle with annotation), median (line in the box), 1st and 3rd quartile (lower and upper border of the box), 1.5× interquartile range (whiskers), and outliers. If some outliers were cut due to *Y*-axis limitation, the maximum is annotated in a red box.

Figure 16 summarizes the basic statistical characteristics of horizontal errors across devices, methods, and conditions. In addition to the means, medians, interquartile ranges (IQR), and outliers shown, a Supplementary Table S1 provides further details including counts, minima, maxima, 95% confidence intervals, and standard deviations (Table S1).

Besides the mean values reported already in previous subchapters, the variability measures (IQR or standard deviations) provide clear distinction between methods and conditions. The SPP method suffers from the highest absolute errors and highest variability. The influence of extreme values can be seen on the difference between means and medians. The “internal” solution provides a better accuracy than SPP in most cases; however, the approaches used by the chipset manufacturers to augment the position are unknown. The SPP method was also the method with the highest difference between the mean horizontal error and the error calculated for average position. The application of precise ephemeris in the PPP method caused a decrease in errors compared to the SPP method, but the errors are still in a range of few meters even under optimal conditions. Here it is necessary to point out that the long observation period (five hours) used in the experiment would not be suitable for practical applications. As expected, the relative positioning using the static method provided the lowest errors and variability.

With regard to conditions, the highest errors and variability were observed for the indoor conditions. These conditions are not typical for the GNSS applications, but the results can facilitate the GNSS usage under conditions where a significant portion of the sky view is covered by obstacles. For methods relying on external corrections (PPP, static), the indoor data was insufficient to provide a solution. Under canopy conditions, errors were ~2–4 times higher compared to the open conditions. This difference was even higher for the static method; however, here these results were affected by an unexplained bias in the N-S direction.

To assess the statistical significance of the differences between devices methods and conditions we employed a three-way ANOVA with post hoc Tukey HSD tests. The ANOVA results are summarized in Table 7.

It was already presumed in the experiment design that the results of differing methods and conditions would be significantly different. This hypothesis was largely confirmed, despite some unexpected cases, such as the internal solution for the Pixel 5 yielding lower errors under canopy than in open conditions. The results for device as a main factor suggest that, if method and conditions are not taken into account, the device itself does not have a significant effect on the mean values. However, the significant two-factor interactions show that the influence of the device on the mean values depends on the method and conditions used (and vice versa). The complex three-way interaction (method–device–condition) was not strong enough to be considered statistically significant at the α = 0.05 significance level (*p* = 0.084).

## 4. Discussion

### 4.1. Quality Control

The qualitative measures of received signal have been a subject of research since the very beginning of the availability of raw GNSS data from Android devices. In this study, we considered have/expected observations ratio, cycle slips, carrier-to-noise density, and multipath.

The have/expected observations ratio describes the completeness of the data. GNUT/Anubis software calculates this ratio based on navigation data and derives the expected number of observations for the particular time and position. This measure is evaluated relatively sparsely compared to, e.g., C/N0 or multipath. For non-flagship smartphones under ideal conditions, Bramanto et al. [45] reported a 70% rate for pseudoranges and 36% for carrier-phase observations. However, contrary to our study, these numbers are based on a comparison with geodetic receiver, not with expected observations based on approximate location and satellite ephemeris. With regard to code observations, the newer cellphone (Pixel 9 Pro XL) provided more complete data with a ratio over 90% in most cases and close to 100% for GPS signals. It is notable that the device maintains the high ratio also under “canopy” conditions, where the Pixel 5 cellphone experiences a drop even higher than 10%. Both devices provided only up to a half of the possible code observations under the “indoor” conditions. This is apparently related to the blocking of the signal reception by the building, which covers approximately 50% of the sky. The have/expected ratio for the carrier-phase observations is significantly lower compared to code observations, with the loss ranging from 10% to almost 25%. The influence of external conditions is visible in all cases, including the Pixel 9 Pro XL where the ratio for code observations was very similar for the “open” and “canopy” conditions. Under these conditions, the newer cellphone provided better completeness of the data. The transition from “open” to “canopy” conditions causes a 10–15% loss of carrier-phase observations. These values cannot be generalized as the conditions under tree vegetation can vary from slightly lighter to a much heavier canopy. The measurements under “indoor” conditions can demonstrate the preference of L5 signals over L1 signals by both the tested devices. While L1 carrier-phase observations achieve only ~10% of their expected values, L5 observations achieve over 40%. This trend, although not so visible, is apparent also under other conditions.

The major reason for discontinuities in observations was the interruptions of signal reception considering two adjacent epochs. Cycle slips, i.e., jumps in the carrier-phase values, represented only a smaller portion of discontinuities. Other authors [3,4,46] recognized another significant reason of discontinuities—so-called duty cycling aimed at prolonging battery life. However, this option was turned off in our case. Considering the total number of carrier-phase observations, the discontinuities in our data represented generally percentages. Even such a relatively small number can cause significant issues especially for positioning methods based on carrier-phase observations. Under “open” conditions, the Pixel 9 Pro XL received the majority of signal with the rate of discontinuities lower than 1%, while the average for the Pixel 5 was over 2%. Under more challenging conditions, the situation is more random, and one device cannot be clearly stated better than the other. L5 signals are more prone to discontinuities as was observed also by Zeng et al. [22] for the Xiaomi Mi 8 phone.

The carrier-to-noise density (C/N0) is amongst the most frequently reported quality measures for smartphone GNSS data. A commonly accepted statement is that smartphones provide C/N0 lower by ~10 dBHz compared to survey-grade devices [31], with the simpler antenna being the main reason [4,47]. In our experiment we did not compare devices of differing grades. Another common observation is that the C/N0 values of smartphone receivers are subject to rapid changes over short time periods [34,46]. The comparison of the tested smartphones in our experiment shows a similar C/N0 for L1 signals. However, for L5 signals the newer device provided C/N0 higher by ~7–8 dBHz under open area conditions. Another comparison of L1 and L5 signals shows that while for the Pixel 5 the C/N0 was higher for L1, it was higher for L5 for Pixel 9 Pro XL. A promising remark is that the BeiDou III signals, available only for the Pixel 9 Pro XL, had a relatively high C/N0 not only under optimal, but also challenging conditions. It is needed to adapt processing software to take full advantage of these relatively new signals. In our study, the carrier-to-noise density was the only QC measure, which regularly decreased in relation to the external conditions from the ideal (“open”) to the most challenging (“indoor”). The results, especially under challenging conditions, cannot be generalized as it is practically impossible to fully replicate the conditions in other test areas. However, other authors studied trends, e.g., under differing degrees of tree canopy and found a positive correlation between the canopy openness and carrier-to-noise density [48].

The multipath effect occurs when the signal does not propagate directly between satellite and the receiver, but it is one or multiple times reflected from the ground or surrounding objects. Urban areas [49] and forests [50] are typical examples of environments with high multipath incidence. The smartphone GNSS receivers have been reported highly susceptible to the multipath. Again, the main reason is a primitive antenna, unable to mitigate the effect [51]. The GNUT/Anubis uses uninterrupted observation periods to calculate the multipath (MP) as a linear combination of code and carrier-phase observations on two frequencies. It is not possible to determine MP in the case of frequent interruptions. This is an issue especially under challenging conditions, where the low number of possible MP calculations leads to high proneness to extremes. Our results demonstrate that the MP is significantly lower for L5 signals compared to L1 signals. The differences between the tested devices are significant only for the Galileo L1 signal, where the Pixel 5 provided clearly better results than the Pixel 9Pro XL. Regarding the measurement conditions, there are multiple cases, where the multipath is higher for “canopy” rather than “indoor” conditions. We explain this by total blocking of signals in the building instead of reflecting the signals, which is more pronounced under tree canopy.

Conclusions of QC analyses suggest that the newer device performed better than the older one, especially regarding the data completeness and signal strength. In the analysis of signal discontinuities and multipath, the devices were comparable, with only rare cases where the Pixel 5 performed better. The advantage of the Pixel 9 Pro XL smartphone si also the ability to receive dual-frequency BeiDou III signals. For the device there is apparent an even stronger inclination to L5 signals, which provide higher signal strength and resilience to the multipath. This can be beneficial for the measurements under conditions adverse to the signal reception, thus allowing more robust solutions for tasks critically related to accurate positioning, e.g., emergency response or autonomous driving. However, GNSS receivers need to be properly designed to take full advantage of L5 signal reception [52].

### 4.2. Positioning

Since 1999 smartphones have provided GNSS-based positioning [53]. However, only in 2016 phones with the Android OS started providing access to raw GNSS data. Until then, the positioning was restricted to an internal receiver solution. This means that the user has access to the final coordinates and some auxiliary information (e.g., number of satellites, signal strength, dilution of precision, estimated accuracy), but cannot directly influence the positioning calculation. However, taking the majority of smartphone users into account, this is a logical and, in most cases, a fully sufficient approach. The internal solution was given appropriate attention by researchers, including studies with multiple tested devices under optimal [25,54] and challenging conditions [18,55,56]. Internal solution typically achieves an accuracy of under 10 m, even close to 2 m under good conditions. Under harsh conditions the variability is higher. In our test, the internal solutions behaved specifically, especially under “open” and “canopy” conditions. For Pixel 5, the internal solution was closest to the reference in the initial phases of the measurement and subsequently continuously drifted several meters. This confirms our previous experience [55]. The Pixel 9 Pro XL did not change the coordinates continuously, but only in incremental steps. This is most probably related to the receiver internal assessment of signal lock robustness as this was not observed under “indoor” conditions. Similar behavior was reported for some Samsung Galaxy devices [25]. Neither of the behaviors is beneficial for the coordinate averaging, which is often the only option of localization improvement when using the internal solution. Based on the results for the internal solution, Pixel 9 Pro XL provided better horizontal accuracy than Pixel 5.

Single-point positioning (SPP) is an absolute positioning method, which uses code observation and derived pseudoranges to determine positions of the receiver. This method is available for all devices with Android 10 and higher. The evaluation, conducted in the RTKLib software, shows a typical random distribution of the positions around the average. The average coordinates for the Pixel 5 were closer to the reference for “open” and “indoor” conditions, with solutions being apparently of lower variability than for the Pixel 9 Pro XL. For the older device, the use of raw code measurements brought an improvement of achieved accuracy compared to the internal solution. However, for the newer device there is no such significant improvement and errors of the average position remain very similar. Comparison with a study from Massarweh et al. [51], where the authors used SPP with data from the Xiaomi Mi8 phone shows that the distribution of errors is in line with our results for Pixel 5, but the variability for Pixel 9 Pro XL is higher. The authors suggested the use of dual frequency measurements, rather than use of multiple systems on single frequency. Psychas et al. [57] achieved sub-meter empirical means for a 14-h observation period using Xiaomi Mi 8 in SPP method. The abovementioned authors agree that rather than traditional filtering based on elevation angle, smartphone data should be filtered based on carrier-to-noise density. One of the reasons is the irregular gain pattern of the smartphone GNSS antenna. Under “canopy” conditions, our results for both devices show a lower mean error, but a higher variability compared to the SPP results reported by Huang et al. [48] for three smartphones in conditions with differing canopy openness.

Precise point positioning is a popular method due to its ability to provide up to centimeter-level positioning accuracy with low demands on the user’s infrastructure, i.e., with a standalone receiver. The CSRS-PPP service used in this study is among the most popular PPP services worldwide. Currently it supports ambiguity resolution (PPP-AR) [58], which enhances the accuracy and robustness of the solution even more. However, a significant limitation of this service for applications with smartphones is its reliance on only GPS/GLONASS L1/L2 signals. It cannot leverage the advantages of observations using Galileo, Beidou systems and L5 signals. Our results suggest only accuracy at the level of a few meters, even with an observation period of five hours. Interestingly, in comparison with SPP results, the benefits of the PPP method are evidently more pronounced for the Pixel 9 Pro XL (horizontal error lowered by 71% under open and 77% under canopy conditions) than for the Pixel 5 (15% open, 5% canopy conditions). These results are modest when compared to the current state-of-the-art. For instance, Li et al. [59] achieved 1 m level accuracy even in real-time kinematic tests. Yi et al. [10] combined data from Galileo High Accuracy Service (HAS) with broadcast ephemerides and achieved an RMS slightly above one meter in a vehicular kinematic test. The authors suggest combining data from all available systems, because the processing with only two GNSS constellations struggles to ensure satisfactory satellite geometry and sufficient observations in realistic user environments. In a static scenario, Li et al. [60] achieved positioning precision of 20 cm considering code-carrier inconsistency in PPP with mixed single- and dual-frequency GNSS observation from a Huawei P40 smartphone.

For the static method, the carrier-phase measurements are necessary. Thus, it is not available for all Android devices, even those providing other raw GNSS measurements. The results are highly dependent on many factors, e.g., base station data [47] and configuration of post-processing software [6]. Our mean horizontal errors reached up to five centimeters for both tested devices under optimal conditions. To increase this level of accuracy, it would be needed to know calibration parameters of used antennas, but these are unknown for tested devices. Under “canopy” conditions, the standard deviations are still under ten centimeters. However, we experienced a ~0.8 m bias in the South direction, which we have no sound explanation for. Magalhães et al. [47] also reported mean errors of ~5 cm for shorter baselines in optimal conditions but also errors up to 30 cm for baselines reaching several hundreds of kilometers. In a previous study [29], we achieved accuracies under 0.5 m, resp. 1.5 m for “open” and “canopy” conditions, using multiple 20 min observations for the Pixel 5 phone. The study has also proven the need to adapt the RTKLib software and its configurations to dealing with smartphone GNSS data. Paziewski et al. [61] used Huawei P30 smartphone and five hours observation period, and achieved centimeter-level accuracy for Fixed solutions, while for Float solution the accuracy was in range of several decimeters. Generally, authors report issues with stable ambiguity fixing and some specific problems (e.g., initial phase bias [46] and long-term drift of phase residuals [61]), which complicate the precise positioning even more.

Generally, the methods, which do not use any corrections (internal, SPP) show a very high level of variability and randomness compared to the methods employing corrections (PPP, static). This could lead to unexpected behavior of mean values (e.g., error for open conditions higher than for canopy conditions), which is hardly explainable by qualitative characteristics of the received signals only. In the case of the internal solution, our study shows that the errors can also be related to device-specific processing approaches, which cannot be directly influenced by the user. The introduction of the filtering and processing approaches from manufacturer’s side is evident also from results, where “internal” solution is in most cases more accurate than SPP. In addition, vertical errors are subject to the well-known multiplication of adverse factors.

Regarding the measurement conditions, most of the studies are conducted under optimal conditions to avoid adverse effects of various obstructions. The positioning accuracy varies intensively based on the applied method. Wu et al. [9] reported an accuracy similar to a geodetic receiver using longer-term static PPP method; however, in a kinematic scenario the difference in trajectories was 3–5 m. Magalhães et al. [47] achieved accuracies under 30 cm using smartphone even with baselines over 100 km, but the accuracy of high-grade GNSS receiver was better. Li et al. [60] tested a Huawei P40 phone in static and kinematic PPP scenario and even with an internal antenna achieved differences of 20 cm and 1 m against a geodetic receiver. In a previous study [29], we examined performance of a Pixel 5 phone using multiple 20 min static seasons and achieved median horizontal errors up to 0.245 m, compared to stable sub-decimeter errors of a u-Blox ZED F9P low-cost, high-accuracy receiver. Under canopy conditions, the smartphone medians deteriorated up to 1.8 m, while the medians for the ZED F9P remained under 10 cm. Purfürst [18] evaluated performance of 10 smartphones and a geodetic receiver in autonomous static scenario under forest canopy and reported DRMSs of smartphones to be 1.4 to 4.5 times higher than for the geodetic receiver. Huang et al. [48] compared three smartphones to a geodetic and recreational-grade GNSS receiver under differing degrees of canopy openness in SPP and PPP mode. They found the smartphone GNSS positioning to be feasible using the PPP method under canopy openness higher than 0.7, but to achieve reasonable accuracy in denser forest stands a geodetic receiver is still necessary. The indoor application of the standalone GNSS positioning is the least typical. Although it is theoretically possible due to high sensitivity GNSS receivers included in smartphones [62], it is highly dependent on the building construction and material. In most cases, GNSS are used only to reference the measurements in the global coordinate system, while the navigation further inside a building is facilitated mostly using GNSS repeaters [63] or various wireless technologies, e.g., Bluetooth, WiFi or ultra-wide band [64,65,66].

### 4.3. Drawbacks, Limitations and Future Challenges

Despite some promising results, our study and the use of raw GNSS data from smartphones, in general, face some limitations and drawbacks, especially from a practical point of view. Firstly, the results cannot be generalized to all Android devices, nor a group of them. This comes from an already mentioned diversity of hardware and software combinations in the Android environment. However, besides the particular results, some trends can be seen. The newer device provided better qualitative characteristics of receiver signals, especially in terms of completeness and carrier-to-noise density. The second trend is the inclination to L5 signals, where the device seems to prefer them over L1 signals. Lastly, the newer device provides access to Beidou III dual-frequency signals. But these technological shifts do not necessarily mean significantly better positioning performance, as can be seen from our statistical analysis. A newer device, even of the same branch/manufacturer, does not automatically mean better GNSS results as reported already by Szot et al. [25]. In general, the smartphone GNSS community could benefit from some standardized experiment designs, which would enable the comparison of devices and positioning approaches on a larger scale. The availability of publicly open datasets is also crucial. Unfortunately, the dataset repository formerly maintained by GNSS Raw Measurement Task Force, is currently unavailable.

The second drawback is the long observation period used in our experiment. Using the period, we achieved an appropriate degree of statistical soundness, but a 5 h observation is hardly typical for an average user or application. The long period could have been split into multiple sessions, but it would distort the characteristic of the measurement, where the highest errors are concentrated in the initial phases of the measurement. From the point of view of methodology, reproducibility and practice, it would be preferable to use multiple shorter observation periods or kinematic measurements in future research design. The majority of typical GNSS uses in smartphones is kinematic and needed in real time [34]. Despite the many attempts and proposed approaches to augment the raw data in real time, e.g., [11,12,22,67,68], we are still not aware of any user-ready application available via official distribution channels (Google Play).

Regarding the GNSS hardware in Android devices, it is often very complicated to obtain any more detailed information reaching beyond the supported systems and frequencies. Especially in integrated hardware setups (like those used in the tested devices), it is difficult to identify the component responsible for GNSS positioning. The situation is even more unclear with regard to used GNSS antennas, which are subject to optimalization mainly due to dimensions constraints., e.g., information about the antenna phase center (which is known only for a very small margin of Android devices) would facilitate precise positioning. Even when the hardware characteristics are specified, the level of hardware integration during the design and manufacturing process is unknown. Thus, in such complex devices as smartphones, even a high-end device with advanced GNSS chips can suffer from poor hardware integration and optimization for high-accuracy GNSS use.

It is commonly agreed that the raw GNSS data from smartphones are of lower quality compared to higher-grade devices. The main hardware-related reason for this questionable GNSS performance is the antenna. Multiple authors have confirmed that when the internal antenna is substituted by a higher-grade external antenna, the quality of the data and availability of positioning solutions increases significantly [15,46,69,70,71]. But this needs serious intervention to smartphone hardware, because smartphones do not include an externally available antenna connector and inclusion of one is of low, if any, priority for manufacturers. A solution for a user with high accuracy and portability demands would be the use of low-cost external GNSS receivers. The market and the application range of low-cost receivers has gradually expanded in recent years [72]. A moderately technically skilled user can achieve a dual-frequency device providing raw GNSS data with a cost up to 100 Euros, with antenna included. More user-friendly sets cost a few hundred Euros. These devices can substitute the internal receiver via wired or wireless connection, with the benefits of external antenna and ability of using the RTK method. The positioning solutions can be sent directly to smartphone via the “Mock location” ability; thus, the use of smartphone-specific applications remains fully available to the user. However, this approach would make the use of raw GNSS data from smartphones obsolete.

## 5. Conclusions

This study highlights the evolution of GNSS performance in Android smartphones, comparing the Google Pixel 5 and Pixel 9 Pro XL under different conditions. While the Pixel 9 Pro XL demonstrated superior signal quality, this did not consistently translate into higher positioning accuracy. The study emphasizes that incremental advancements in hardware alone are insufficient for significant improvements in GNSS accuracy—software optimization and processing techniques play crucial roles. Moreover, it is close to impossible to generalize the results due to the high hardware and software diversity of Android devices. To facilitate reproducibility and the possibility of applying other processing approaches, a full dataset of raw GNSS measurements is provided openly.

The results indicate that dual-frequency and multi-constellation capabilities contribute to better GNSS performance, particularly under suboptimal conditions. However, the increased signal availability does not necessarily lead to higher precision, as seen in the discrepancies in single-point positioning (SPP) and static positioning results. One of key takeaways is the growing importance of L5 signals, which consistently provide higher signal strength and lower multipath errors. This suggests that future GNSS-enabled smartphones should prioritize L5 support for improved positioning reliability. Additionally, the findings highlight the need for real-time processing enhancements and refined algorithms to fully exploit raw GNSS data and shift them from research to user-ready applications.

Future research and development should focus on improving software-based filtering techniques (e.g., C/N0 rather than elevation based), optimizing antenna design and documenting its properties to enable high accuracy, and exploring the potential of low-cost external GNSS receivers as an alternative for high-precision applications. The integration of alternative sources of corrections (e.g., SBAS, Galileo HAS, EGNOS, precise ephemeris, etc.) can also improve the positioning performance. Finaly, exploiting other smartphone capabilities like inertial measurement sensors, wireless accessibility, cloud-based solutions and real-time online calculations is also proving to be beneficial. As GNSS technology in smartphones continues to evolve, a balanced approach combining hardware advancements with adapted software solutions will be essential to achieving significant accuracy improvements and practical applicability.

## Figures and Tables

**Table 1:** Basic GNSS-related characteristics of tested devices.

Device	Pixel 5	Pixel 9 Pro XL
GNSS hardware name	redfin;MPSS.HI.2.0.c8-00264-SAIPAN_GEN_PACK-1.34428.9 (Qualcomm Snapdragon 765G)	S.LSI,K042,SPOTNAV_4.15.1_18_240628_R1_225564 (Samsung Exynos 5400)
Hardware year	2018	2023
Supported GNSS constellations and frequencies	GPS (L1, L5) GLONASS (L1) Galileo (E1, E5a) BeiDou (B1I)	GPS (L1, L5) GLONASS (L1) Galileo (E1, E5a) BeiDou (B1I, B1C, B2a)

**Table 2:** Mean horizontal errors (HEs), vertical errors (VEs), and errors of the average position (AP) acquired by the internal solution for Pixel 5 and Pixel 9 Pro XL.

	Pixel 5			Pixel 9 Pro XL		
Condition	HE (m)	AP (m)	VE (m)	HE (m)	AP (m)	VE (m)
Open	6.56	6.54	6.38	1.66	1.64	−0.88
Canopy	3.39	3.34	0.32	2.77	2.77	4.98
Indoor	14.20	14.15	−11.46	11.69	9.81	−12.14

**Table 3:** Mean horizontal (HE), vertical (VE) errors and the error of the average position (AP) acquired by the SPP method for Pixel 5 and Pixel 9 Pro XL.

	Pixel 5			Pixel 9 Pro XL		
Condition	HE (m)	AP (m)	VE (m)	HE (m)	AP (m)	VE (m)
Open	3.85	0.56	0.12	5.47	1.68	−0.17
Canopy	10.00	6.44	8.38	10.80	2.93	13.90
Indoor	220.31	8.28	6.44	175.02	9.81	−2.94

**Table 4:** Mean horizontal (HE), vertical (VE) errors and the error of the average position (AP) acquired by the PPP method for Pixel 5 and Pixel 9 Pro XL.

	Pixel 5			Pixel 9 Pro XL		
Condition	HE (m)	AP (m)	VE (m)	HE (m)	AP (m)	VE (m)
Open	3.28	2.76	−0.10	1.61	1.58	1.55
Canopy	9.47	8.70	20.70	2.46	2.34	16.96
Indoor	-	-	-	11.14	7.44	18.36

**Table 5:** Positioning errors and solution types for Pixel 5 and Pixel 9 Pro XL under optimal conditions.

Device	HE (m)	AP (m)	VE (m)	Fix/Float Ratio (%)	Std (m)
Pixel 5	0.047	0.050	−0.120	37.5/62.5	0.011
Pixel 9 Pro XL	0.042	0.050	−0.019	82.1/17.9	0.009

**Table 6:** Positioning errors and solution types for Pixel 5 and Pixel 9 Pro XL under canopy conditions.

Device	HE (m)	AP (m)	VE (m)	Fix/Float/DGNSS Ratio (%)	Std (m)
Pixel 5	0.826	0.820	0.323	24.8/71.5/3.7	0.054
Pixel 9 Pro XL	0.879	0.873	−1.119	13.4/84.8/1.8	0.025

**Table 7:** Results of 3-way ANOVA considering devices, methods, and conditions. Main effects and their interactions with significant differences (*p* < 0.05) are marked (*).

	df	sum_sq	mean_sq	F	PR(>F)
C(method)	3.0	2.894590 × 10^8^	9.648632 × 10^7^	162.501442	2.855157 × 10^−105^ *
C(device)	1.0	1.939542 × 10^6^	1.939542 × 10^6^	3.266561	7.070651 × 10^−2^
C(conditions)	2.0	3.671323 × 10^8^	1.835661 × 10^8^	309.160531	6.994686 × 10^−135^ *
C(method):C(device)	3.0	6.761830 × 10^6^	2.253943 × 10^6^	3.796072	9.802334 × 10^−3^ *
C(method):C(conditions)	6.0	4.398505 × 10^8^	7.330842 × 10^7^	123.465419	1.367281 × 10^−156^ *
C(device):C(conditions)	2.0	5.706611 × 10^6^	2.853305 × 10^6^	4.805513	8.185011 × 10^−3^ *
C(method):C(device):C(conditions)	6.0	6.609575 × 10^6^	1.101596 × 10^6^	1.855298	8.439085 × 10^−2^
Residual	371,938.0	2.208407 × 10^11^	5.937567 × 10^5^	NaN	NaN

## Data Availability

The experimental dataset, including raw GNSS data from smartphones, base data, RTKLib configuration, and reference coordinates is available at Mendeley Data, DOI: 10.17632/b6rnphwxnv.

## Supplementary Materials

The following supporting information can be downloaded at: https://www.mdpi.com/article/10.3390/s25144452/s1, Table S1: Summary of statistical characteristics according to devices, conditions and positioning methods.

## Abbreviations

The following abbreviations are used in this manuscript:

GNSS	Global Navigation Satellite Systems
SPP	Single-Point Positioning
LBS	Location-Based Services
C/N0	Signal-to-noise density
MP	Multipath
ETRS	European Terrestrial Reference System
ETRF	European Terrestrial Reference Frame
ITRF	International Terrestrial Reference Frame
QC	Quality Control
OS	Operating System
SBAS	Satellite-Based Augmentation Systems
CORS	Continuously Operating Reference Station
SKPOS	Slovak Real-Time Positioning Service
ADR	Accumulated Delta Range
DGNSS	Differential GNSS
RTK	Real-Time Kinematic
PPP	Precise Point Positioning

## Appendix A

Skyplots with cycle slips occurrence according to devices and signal frequencies under “canopy” and “indoor” conditions. Red ticks represent “Lost Lock Indicator” (LLI) flags extracted from RINEX files. Obstructed areas are shaded grey.

## Appendix B

Skyplots with interpolated values of carrier-to-noise density (C/N0) according to devices and signal frequencies under “canopy” and “indoor” conditions. Coefficiens of determination between C/N0 and elevation angle. Obstructed areas are shaded grey.
